# Transcriptomics and methylomics in chronic periodontitis with tobacco use: a pilot study

**DOI:** 10.1186/s13148-017-0381-z

**Published:** 2017-08-10

**Authors:** Young-Dan Cho, Pil-Jong Kim, Hong-Gee Kim, Yang-Jo Seol, Yong-Moo Lee, Young Ku, In-Chul Rhyu, Hyun-Mo Ryoo

**Affiliations:** 1 Department of Periodontology, School of Dentistry, Seoul National University, 101 Daehak-no, Jongno-gu, Seoul, 03080 South Korea; 2 Department of Molecular Genetics, School of Dentistry, Seoul National University, 1 Gwanak-ro, Gwanak-gu, Seoul, 08826 South Korea; 3 Department of Dental Services Management and Informatics, School of Dentistry, Seoul National University, 1 Gwanak-ro, Gwanak-gu, Seoul, 08826 South Korea

**Keywords:** DNA methylation, Epigenomics, Extracellular matrix, Periodontal disease, Smoking, Transcriptome

## Abstract

**Background:**

Accumulating evidence suggests that tobacco smoking affects the susceptibility to and severity of chronic periodontitis. Epigenetics may explain the role of smoking in the development and progress of periodontal disease. In this study, we performed transcriptomic and methylomic analyses of non-periodontitis and periodontitis-affected gingival tissues according to smoking status.

**Methods:**

Human gingival tissues were obtained from 20 patients, including non-smokers with and without periodontitis (*n* = 5 per group) and smokers with and without periodontitis (*n* = 5 per group). Total RNA and genomic DNA were isolated, and their quality was validated according to strict standards. The Illumina NextSeq500 sequencing system was used to generate transcriptome and methylome datasets.

**Results:**

Comprehensive analysis, including between-group correlation, differential gene expression, DNA methylation, gene set enrichment, and protein-protein interaction, indicated that smoking may change the transcription and methylation states of extracellular matrix (ECM) organization-related genes, which exacerbated the periodontal condition.

**Conclusions:**

Our results suggest that smoking-related changes in DNA methylation patterns and subsequent alterations in the expression of genes coding for ECM components may be causally related to the increased susceptibility to periodontitis in smokers as they could influence ECM organization, which in turn may have an effect on disease characteristics.

**Electronic supplementary material:**

The online version of this article (doi:10.1186/s13148-017-0381-z) contains supplementary material, which is available to authorized users.

## Background

Periodontal diseases are typical inflammatory conditions caused by bacterial infection and promoted by environmental factors or other modifying factors [1]. Chronic periodontitis presents a destructive periodontal disease that leads to alveolar bone resorption [2]. Tobacco smoking is considered a major risk factor, and many studies have demonstrated that smoking alters the development and progression of periodontitis [3–5]. Other risk factors that modify the host response to the challenge of bacterial infection and may induce periodontitis are alcohol, diet, pollution, and drugs [6].

An increasing number of recent studies have focused on the role of epigenetic events in the development of various diseases [7, 8]. Unlike genetics which analyzes changes in the DNA sequence, epigenetics represents the study of cellular or physiological phenotypic trait variations caused by environmental or external factors, which modulate gene expression without altering DNA sequence [9]. Epigenetic modifications include chemical alteration of DNA and associated proteins such as histones, which leads to chromatin remodeling and plays an important role in regulating gene expression [10]. Among these effects, DNA methylation is a common epigenetic mechanism observed in human cells [11]. DNA methylation carried out by DNA methyltransferases typically occurs in CpG dinucleotide-rich regions termed “CpG islands,” which are mainly located in gene promoters, and is associated with gene silencing [12]. The methylated sites interact with the methyl-CpG-binding domain proteins (MBDs) which in turn recruit histone deacetylase-containing complexes and induce histone condensation. Moreover, histones can also be directly modified by methylation. Both mechanisms block the binding of transcription factors to gene loci; however, while histone modification is transient, DNA methylation exhibits a more stable nature of gene regulation [13].

Some reports have suggested that the CpG methylation status of inflammation-related genes (e.g., *IL-2* and *IL-8*) is implicated in gene expression in chronic periodontitis [12, 14, 15]. Most epigenetic studies of periodontitis used low-throughput-level screening and were focused on host response to bacterial infection [16, 17], while the impact of environmental or external factors on DNA methylation was somewhat neglected. The aim of this study was to test a hypothesis that tobacco smoking could change the epigenetic state of periodontal cells and modulate the susceptibility to or severity of periodontitis. To determine whether this is the case, we performed comprehensive genome-wide high-throughput analysis using RNA sequencing (RNA-seq) combined with reduced representation bisulfite sequencing (RRBS) in smokers and non-smokers with and without periodontitis.

## Methods

### Patient selection

The study population included 20 generally healthy patients without and with chronic periodontitis (Table 1). The smoker group (*n* = 10) comprised people who smoked for at least 5 years; individuals who had never smoked were included in the non-smoker group (*n* = 10). Statistical analyses were performed using SPSS 12.0 (SPSS Inc., Chicago, IL, USA). For analysis of patient age and smoking condition, a two-tailed Wilcoxon rank sum test was conducted. For analysis of sex, two-tailed Fisher’s exact test was used. Visual inflammation signs, probing pocket depth (PPD), bleeding on probing (BOP), and alveolar bone resorption in radiography were used to diagnose periodontitis. Gingival tissue of patients without any clinical inflammatory signs, BOP, and alveolar bone loss, and with PPD ≤ 4 mm served as control (non-periodontitis group). Chronic periodontitis was determined by the presence of chronic inflammatory signs, PPD ≥ 6 mm, BOP, and alveolar bone loss (chronic periodontitis group). Exclusion criteria were acute or aggressive periodontitis, any severe systemic disease that could affect the periodontal condition, current pregnancy or lactation, and the use of systemic antibiotics or anti-inflammatory medications within 6 months prior to baseline.

**Table 1:** Background of the study population

	Non-smoker			Smoker			*P*
	Non-periodontitis NN, (*N* = 5)	Periodontitis NP, (*N* = 5)	*P*	Non-periodontitis SN, (*N* = 5)	Periodontitis SP, (*N* = 5)	*P*	Non-smoker vs. Smoker
Age (years)	44 (41–55)	53 (52–55)	0.525^*^	52 (47–54)	56 (52–56)	0.205^*^	0.676^*^
Men:women	3:2	1:4	0.444^†^	5:0	4:1	0.206^†^	0.350^†^
Cigarettes/day	–	–	–	17.5 (5–25)	20.5 (8–22)	0.917^*^	–
Years smoking	–	–	–	16.5 (16.5–24)	27 (17–27.5)	0.295^*^	–

Values are median (interquartile range)

^*^ *P* values are calculated by two-tailed Wilcoxon rank sum test

^†^ *P* values are calculated by two-tailed Fisher’s exact test

### Collection of gingival tissue samples

Gingival tissues were resected from maxillary or mandibular molar sites in the process of crown lengthening for the non-periodontitis group or open flap debridement for the chronic periodontitis group. All biopsy sites were subjected to surgical procedures for the first time, and the specimens were obtained by the same experienced periodontal surgeon. After local anesthesia, buccal and palatal/lingual scalloped internal incision was made around the teeth away from the free gingival margin. The size of collected gingival sample was approximately 0.5 × 1.0 cm, which included the epithelium and connective tissue. Tissues were immediately transferred to the laboratory, where half of each sample was used for DNA and another half for RNA isolation.

### RNA isolation, quality control, and sample preparation for RNA-seq

Total RNA was isolated from gingival tissue using the RNeasy®-Mini kit (Qiagen, Valencia, CA, USA). RNA quality was evaluated using the RNA 6000 NanoLabChip Kit for the Bioanalyzer system (Agilent Technologies, Santa Clara, CA, USA). All samples had an RNA integrity value > 8. A cDNA library was generated for each sample, and its quality was evaluated using the Bioanalyzer system.

### DNA isolation, quality control, and sample preparation for reduced representation bisulfite sequencing (RRBS)

Genomic DNA (gDNA) was isolated from gingival tissue using the DNeasy® Blood & Tissue Kit (Qiagen), and its quality was evaluated using a NanoDrop spectrophotometer (NanoDrop Technologies, Wilmington, DE, USA). The ratio of the absorbance at 260 and 280 nm (OD260/280) was used as an indicator of sample purity: DNA was considered pure at OD260/280 of 1.8–2.0. For additional quality check, agarose gel electrophoresis was performed to exclude such effects as RNA interference and DNA nicking or damage. Half of each gDNA sample was subjected to bisulfite conversion using the EpiTect® Bisulfite kit (Qiagen).

### RNA sequencing

In total, 10 non-periodontitis and 10 chronic periodontitis samples yielded a sufficient amount of RNA (300 ng) for subsequent analysis. RNA sequencing libraries were prepared using the Illumina TruSeq RNA library kit, and paired-end sequencing with 100 bp reads was performed using the Illumina Nextseq500 platform, resulting in 46.1 M reads per sample in average. For removing adapters, and trimming and quality control, we used Trimmomatic [18]. PE-phred33 ILLUMINACLIP:TruSeq3-PE.fa:2:30:10 MINLEN:75 2. STAR15 [19] was utilized to align the fastq files to the human GRCh37/hg19 reference genome with the following settings: maximum intron size, 500 kb; minimum intron size, 20; four mismatches allowed. HTSeq (version HTSeq-0.6.1) [20] was performed to produce raw counts.

### Comparison of gene expression profiles between groups

To assess the homogeneity of gene expression profiles between groups, Spearman’s rank correlation coefficient (SRCC) of log-transformed gene expression for each sample was calculated using the cor function in R (http://www.r-project.org). The median and interquartile ranges of SCC for all between-group comparisons were calculated, and scatter plots of the worst SRCC in each group were obtained as representative plots. SRCC values were compared by Wilcoxon rank sum test, and *P* < 0.05 was considered statistically significant.

### Differential expression analysis using DESeq2

DESeq2 in the R-package was used to identify differentially expressed (DE) genes in each condition [21]. In DESeq2, the Wald test specifically developed for raw RNA-seq count data with a negative binomial model was used to identify differentially expressed genes. Genes with *P* < 0.05 were used for further enrichment analysis.

### DNA methylation analysis

The gDNA methylation profiles were analyzed using reduced representation bisulfite sequencing. Briefly, we treated gDNA with sodium bisulfite using the EpiTech® Bisulfite kit (Qiagen), digested with the *Msp*I restriction enzyme, and selected fragments averaging 100 to 250 bp. We multiplexed four samples per lane and sequenced the libraries using single-end 100-bp reads and the Illumina Nextseq500 platform; as a result, an average of 25.1 M reads per sample were obtained. We performed initial quality control for fastq files using FastaQC. The reads were aligned using the Bowtie2 end-to-end alignment mode allowing four mismatches; hg19 was used as a reference genome [22]. BS-seeker2 [23] was used for calling the methylation status of the individual CpG sites. The resultant text files of BS-seeker2 were cleaned up to obtain the methylation percentage of individual CpG sites for each case. In a 1000-bp window (from + 500 to − 500 bp) based on transcription start sites of all genes identified using RNA-Seq, CpG methylation percentages were obtained to assess the effect of methylation near transcription start sites on gene expression. For the non-smoker/non-periodontitis (NN), non-smoker/periodontitis (NP), smoker/non-periodontitis (SN), and smoker/periodontitis (SP) groups (Table 1), the statistical significance of CpG methylation for each gene was calculated to identify statistically significant methylation near gene loci. Genes with *P* < 0.05 were used for further enrichment analysis.

### Correlation between methylation and gene expression

To analyze the relationship between methylation and expression of genes that showed statistically significant differences in both parameters, regression analysis was performed. To determine whether differential DNA methylation occurs in subjects with periodontitis compared to normal subjects, a regression model with methylation (*x*_*i*1_) and periodontitis (*x*_*i*2_) as the predictor variables and gene expression (*y*_*i*_) as the response variable was fitted by R function aov:

$$ {y}_i={\beta}_0+{\beta}_1{x}_{i1}+{\beta}_2{x}_{i2}+{\beta}_3{x}_{i1}{x}_{i2}+{\epsilon}_i $$

where *i* represents each gene. This model was compared to the null linear model having only methylation (*x*_*i*1_) as the predictor variable:

$$ {y}_i={\beta}_0+{\beta}_1{x}_{i1}+{\epsilon}_i $$

An analysis of variance (ANOVA) of the two models was conducted to elucidate the effect of periodontitis on the regression model.

### Gene set enrichment analysis

To analyze biological significance of the obtained methylation data, we performed gene set enrichment analysis (GSEA) of the Gene Ontology Biological Processes (GO-BP). The GO term source to calculate GSEA was obtained using the GO.db library (version 2.1) of R. The GSEA test of each group was performed by manipulating the clusterProfiler library of R [24]. Gene sets with *q* < 0.05 were considered to be statistically significant.

### Protein-protein interaction

Based on the assumption that the products of methylated genes might directly or indirectly interact with other proteins, we obtained a list of genes associated with protein-protein interaction (PPI) by performing PPI analysis for DE genes exhibiting DNA methylation in non-smoker and smoker cases. We extracted PPI-affected genes in the database from iRefIndex 13.0 [25] by manipulating the iRefR library which bridges the iRefIndex database to an R environment. The gene list obtained by PPI was analyzed by GSEA to identify gene sets with potential relation to both DE genes and DNA methylation.

## Results

### Study participants

Twenty patients, 13 men and 7 women, were included in this study (Table 1). The median age of non-smokers in the NN and NP groups was 44 and 53 years, respectively, and that of smokers in the SN and SP groups was 52 and 56 years, respectively. In general, there were more men than women in the smoker group (*P* = 0.350), and patients in the periodontitis group were slightly older compared to the healthy group (*P* = 0.676), but the difference was not significant. Furthermore, the mean number of cigarettes per day and smoking duration tended to be lower in the SN group compared to the SP group, but the difference was not significant (*P* = 0.917 and *P* = 0.295, respectively).

### Correlation of gene expression with smoking

**Fig. 1:**
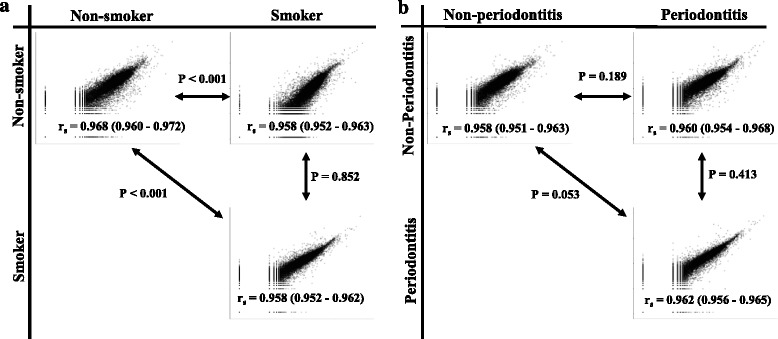
Representative correlation plots for mRNA expression. **a** Non-smoker vs. smoker groups. **b** Non-periodontitis vs. periodontitis groups. The *r*_s_ values in the scatter plot are shown as the median and interquartile of Spearman’s rank correlation coefficients between the cases of the groups. *P* values near the line with arrows at both ends are *P* values determined by the Student Wilcoxon rank sum test to compare the Spearman rank correlation coefficients between two groups shown in the *x* and *y* axes

In the correlation analysis of the RNA-seq data, we considered smoking (Fig. 1a) and periodontal condition as factors (Fig. 1b). When smoking status was considered, the median SRCC between the two non-smoker groups (0.968) was higher than that between the two smoker groups (0.957) or between non-smokers and smokers (0.958) (Fig. 1a). When Wilcoxon rank sum test was performed for each comparison correlation values, comparisons between the correlation of the non-smoking group and that of other groups were found to be statistically significant (*P* < 0.01). When periodontal status was considered (Fig. 1b), the two periodontitis groups exhibited the highest mean correlation coefficient (0.962). In the Wilcoxon rank sum test, no group comparison was found to be statistically significant.

### Differential gene expression pattern in periodontitis with a smoking factor

**Fig. 2:**
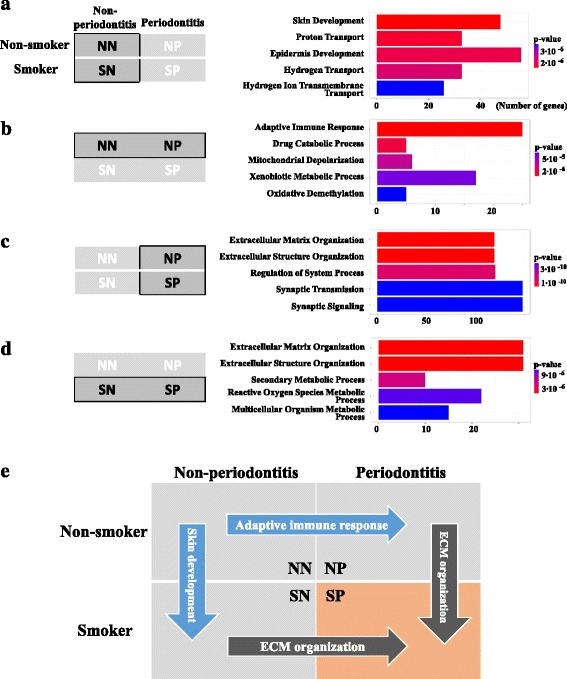
Gene Ontology Biological Processes (GO-BP) term enrichment analysis. **a** Non-smoker/non-periodontitis (NN) vs. smoker/non-periodontitis (SN). **b** NN vs. non-smoker/periodontitis (NP). **c** NP vs. smoker/periodontitis (SP). **d** NS vs. SP. *Left boxes* show the group used for the GO enrichment analysis. *Right horizontal bar charts* show enrichment analysis performed using clusterProfiler. The top five enriched GO-BP categories are listed. The *P* values are sorted by significance (low, blue; high, red). The *bar length* represents the observed number of genes in the experimental set within the respective KEGG pathway. **e** Summary plot for the most significant GO-BP terms in each group

The number of DE genes without controlling for the false discovery rate (FDR) identified between NN and SN cases was 1841 (Additional file 1: Table S1). Among the DE genes, 1105 were upregulated and 736 were downregulated in the SN group. In comparison, 2901 DE genes without controlling for the FDR were observed between NP and SP cases (Additional file 1: Table S2); among them, 1298 were upregulated and 1603 were downregulated in the SP group. In the NN and SN groups, statistically significant DE genes were enriched in the GO-BP categories of “skin development” (GO:0043588, *P* = 1.23E^−06^, *q* = 2.54 E^−03^; e.g., *Staphylococcus aureus* infection, basal cell carcinoma, or melanoma) (Fig. 2a), whereas in the NN and NP groups, DE genes were mostly enriched in the category “adaptive immune response” (GO:0002250, *P* = 1.70E^−05^, *q* = 4.48 E^−02^; e.g., natural killer cell-mediated cytotoxicity) (Fig. 2b). Notably, in smokers, the genes associated with “ECM organization” (GO:0030198) were increased in periodontitis (SP compared to NP or SN, *P* = 1.61E^−16^ and *P* = 4.97E^−07^, *q* = 2.95E^−11^ and *q* = 9.33E^−04^, respectively; Fig. 2c, d). Collectively, these results show the differential gene expression pattern with the smoking effect in different periodontal conditions (Fig. 2e).

### Methylation analysis and combined analysis of transcriptome and methylome data

**Fig. 3:**
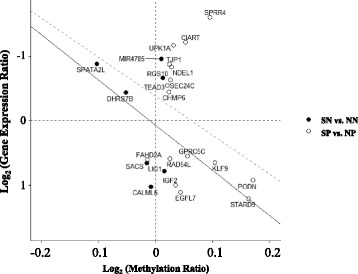
Scatter plot of DNA methylation versus gene expression. The *x* and *y* axes present the log_2_-scaled ratios of DNA methylation and gene expression, respectively. *Solid and dashed lines* indicate the regression of smoker/periodontitis (SP) vs. smoker/non-periodontitis (SN) and of SN vs. non-smoker/non-periodontitis (NN)

The number of genes that were differently methylated (DM) in a 500-bp window of the transcription start site between NN and SN cases was 84 when FDR control was not used. Among the DM sites, 36 were hyper-methylated and 48 were hypo-methylated in SN (Additional file 1: Table S3). The number of DM sites between NP and SP cases was 96 when no FDR control was conducted; among them, 30 were hyper-methylated and 66 were hypo-methylated in SP (Additional file 1: Table S4). The numbers of DE and DM genes were 7 and 16, respectively, without overlap (Additional file 1: Table S5). The regression model of methylation and expression of genes had a negative slope (− 4.17) without a significant difference in the model with periodontitis as a response variable (*P* = 0.684). Among the DE and DM genes showing a negative slope, three genes (*DHRS7B*, *LIG1*, and *SPATA2L*) were identified between NN and SN cases and seven genes (*EGFL7*, *GPRC5C*, *IGF2*, *KLF9*, *PODN*, *RAD54L*, and *STARD9*) were identified between NP and SP cases (Fig. 3).

### PPI analysis

**Fig. 4:**
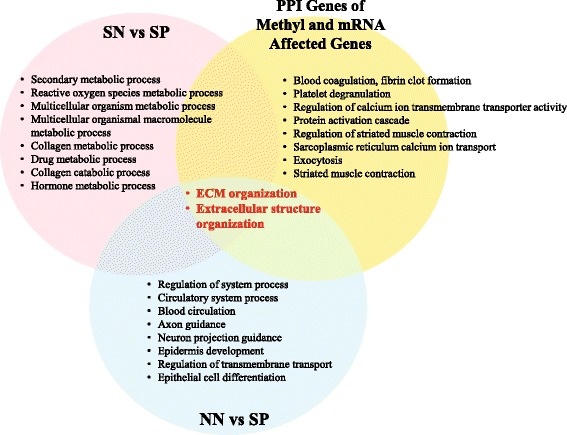
Venn diagram of the top 10 gene ontology biological terms for smoker/non-periodontitis (SN) vs. smoker/periodontitis (SP) and non-smoker/non-periodontitis (NN) vs. SP, and PPI analysis of genes with differential methylation and mRNA expression. ECM organization and extracellular structure organization are common terms in SN vs. SP (*pink circle*), NN vs. SP (*blue circle*), and among PPI of the genes with differential methylation and mRNA expression (*yellow circle*). No common gene ontology terms were identified between the two circles

PPI analysis performed for 17 genes exhibiting inverse correlation of DNA methylation with gene expression between non-smokers (NN and NP) and smokers (SN and SP) (Additional file 1: Table S6) identified 157 genes as potentially affected by PPI (Additional file 1: Table S7). Among them, 50 genes were differentially expressed and 107 genes showed no change between the non-smoker and smoker groups (Additional file 1: Table S7). Fisher’s exact test indicated that this ratio was statistically significant (*P* = 0.040). Among the GO-BP terms of PPI-affected genes (Fig. 4, yellow circle), “blood coagulation and fibrin clot formation” was the most significantly enriched (GO:0007596, *P* = 1.76E^−10^ *, q* = 4.04E^−07^). The top 10 GO-BP terms of PPI-affected genes (Fig. 4, yellow circle) in common with DE genes for SN vs. SP (Fig. 4, pink circle) or NN vs. SP (Fig. 4, blue circle) were “ECM organization” (GO:0030198, *P* = 6.45E^−08^ *, q* = 2.57E^−05^) and “extracellular structure organization” (GO:0043062, *P* = 6.73E^−08^ *, q* = 2.57E^−05^) (Fig. 4). The top 10 GO-BP terms of PPI-affected genes did not overlap with those of NN vs. NP.

## Discussion

To the best of our knowledge, this is the first high-throughput study investigating both DNA methylation and gene expression to evaluate the effect of smoking on chronic periodontitis. Our results indicate that smoking is closely associated with ECM organization-related genes, which intensify the periodontal condition as indicated by DE and DM patterns, and PPI-affected genes. As smoking might contribute to periodontal disease susceptibility through microbial infection [26–28] and the development of malignancy [29], many variations of mRNA expression associated with a different DNA methylation status might be important in the context of biological and clinical aspects of this disease.

By correlation analysis, we revealed statistically significant differences between the groups depending on the smoking status, when non-smokers vs. non-smokers exhibited the highest Spearman’s rank correlation coefficient value (Fig. 1a). The difference in SRCC can be explained by changes in gene expression caused by smoking (e.g., years of smoking or number of cigarettes smoked per day) [30, 31], gender-related variations [32], or personal habits [33]. Different internal and external stress factors may trigger cellular responses which may result in the damage of macromolecular structures and functions, promoting a variety of pathological conditions [34].

Periodontitis is a chronic inflammatory disease leading to the destruction of periodontal tissues, including alveolar bone [35]. Previously, De Souza et al. [14] have reported variations in DNA methylation of immunity-related genes between healthy individuals and periodontitis patients [14]. Consistent with these results, our RNA-seq data indicated that the GO-BP category related to adaptive immune response are highly represented; however, the difference in the methylation status between healthy and periodontitis group was not significant (Fig. 2b). One of the important host factors in periodontitis is the activity of matrix metalloproteinases, which are responsible for collagen and ECM degradation of periodontal tissues [36]. Consistent with this notion, we revealed differential transcriptional regulation of ECM-related genes in the periodontitis group (Fig. 2) and changes in the DNA methylation pattern in the smoker group (Fig. 3). Notably, mRNA expression analysis indicated that the GO-BP term description of NP vs. SP (Fig. 2c) and SN vs. SP (Fig. 2d) identified the same term, ECM organization, indicating the expression, assembly, and arrangement of ECM constituents according to the web application AmiGO [37]. Smoking may exert a variety of biological effects on the ECM [35, 38], which may account for the enrichment of this GO-BP term by smoking in periodontitis patients (from NP to SP) as well as by periodontitis in smokers (from SN to SP) (Fig. 2e). Methylation analysis indicated that smoking affected both DNA methylation and gene expression, which showed negative correlation (Fig. 3). Several novel biomarkers for smoking-associated blood DNA methylation have been identified using the Illumina 27K array [39, 40] and Illumina Infinium HumanMethylation 450K BeadChip array [41]. Among them, seven CpGs (F2RL3 [cg03636183], AHRR [cg21161138 and cg05575921], 2q37.1 [cg21566642, cg01940273, and cg05951221], and 6p21.33 [cg06126421]) were common among most DM sites [42]. However, we could not identify common biomarkers based on our data (Additional file 1: Table S3 and S4), which can be due to low sample size and the fact that we used gingival tissue. We assume that the changes in DNA methylation that affect gene expression were localized to positions near transcription start sites; however, methylation near transcription start sites could not explain all epigenetic aspects, suggesting the need for further studies recommended in the “Encyclopedia of DNA Elements (ENCODE)” project, which aims to identify comprehensive functional elements in the human genome.

The GO terms ECM organization and extracellular structure organization were also found to be common among SN vs. SP and NN vs. SP groups and among genes that had PPI relationship with methylated DE genes (Fig. 4). However, among the GO terms identified through the combined analysis of transcriptome and methylome data for NN vs. SN or NP vs. SP, no common GO terms were obtained by comparing NN vs. NP or SN vs. SP. PPI extends the effect of the identified DE and DM genes to that of indirectly related genes through protein-protein cooperation. In this study, PPI broadened our perspective from single genes to more complex functional units (Fig. 4). The category showing the greatest PPI effect, blood coagulation and fibrin clot formation, is considered to be associated with wound healing in periodontal disease (Fig. 4, yellow circle) [43]. Although based on these results, we cannot conclude that DNA methylation caused by smoking directly affects the periodontitis status; the genes identified as related to those with increased DNA methylation may disturb ECM organization through PPI, which in turn would affect periodontitis in smokers.

Collectively, it is conceivable that both smoking-related DNA methylation and gene expression profiles reflect an increased sensitivity of periodontitis patients to environmental hazards such as smoking. Our results suggest that the smoking factor may contribute to weakening of periodontal tissue healing potential and accelerate tissue destruction through effects on DNA methylation and expression of ECM organization-related genes. Although DNA methylation caused by smoking may not directly affect periodontitis, the genes regulated by smoking-mediated epigenetic modifications can influence ECM organization, which in turn may have an impact on the disease characteristics.

Some genes were differentially expressed between groups; however, the conclusions may be premature because of study limitations such as small sample size and other unaccounted epigenetic factors (histone modification, microRNA, etc.). In this study, we could not identify specific target genes based on DNA methylation and mRNA expression patterns, as the results did not reach statistical significance. Instead, we detected the trend of gene expression and methylation shifts depending on smoking and periodontitis statuses by analyzing the correlation differences between groups, putative functions of DE/DM genes based on GO-BP terms, and protein-protein interactions. Our study provides a foundation for further studies using larger cohorts and incorporating a broader spectrum of epigenetic modifications, which are required to address potential mechanisms underlying the observed patterns and to discover diagnostic biomarkers for the susceptibility of patients with periodontitis to environmental hazards.

## Conclusions

In summary, our data indicate that smoking may negatively affect the healing potential of periodontal tissue and accelerate its destruction through effects on DNA methylation and expression of ECM organization-related genes.

## Additional file

Additional file 1: Table S1. Differentially expressed (DE) genes in SN vs. NN. **Table S2.** Differentially expressed (DE) genes in SP vs. NP. **Table S3.** Differentially methylated (DM) genes in SN vs. NN. **Table S4.** Differentially methylated (DM) genes in SP vs. NP. **Table S5.** Differentially expressed (DE) and methylated (DM) genes in smokers vs. non-smokers. **Table S6.** Differentially expressed (DE) and methylated (DM) genes. **Table S7.** Genes involved in protein-protein interaction (PPI). (XLSX 309 kb)

## Abbreviations

BOP: Bleeding on probing
DE: Differentially expressed
DM: Differentially methylated
ECM: Extracellular matrix
FDR: False discovery rate
GO-BP: Gene Ontology Biological Processes
GSEA: Gene set enrichment analysis
PPD: Probing pocket depth
PPI: Protein-protein interaction
RNA-seq: RNA sequencing
RRBS: Reduced representation bisulfite sequencing
SRCC: Spearman’s rank correlation coefficient
